# Unraveling the Adsorption Behavior of Thymol on Carbon and Silica Nanospheres for Prolonged Antibacterial Activity: Experimental and DFT Studies

**DOI:** 10.1021/acsabm.3c00460

**Published:** 2023-09-27

**Authors:** Narongrit Sosa, Jakkapop Phanthasri, Nuttapon Yodsin, Yodsagon Samun, Chompoonut Rungnim, Supawadee Namuangruk, Saran Youngjan, Wanwitoo Wanmolee, Teera Butburee, Hideki Nakajima, Ratchadaporn Supruangnet, Kajornsak Faungnawakij, Pongtanawat Khemthong, Suchada Sukrong

**Affiliations:** † Functional Materials and Nanotechnology Center of Excellence, Walailak University, Nakhon Si Thammarat 80160, Thailand; ‡ National Nanotechnology Center (NANOTEC), National Science and Technology Development Agency (NSTDA), Pathum Thani 12120, Thailand; § Department of Chemistry, Faculty of Science, Silpakorn University, Nakhon Pathom 73000, Thailand; ∥ Center of Excellence in DNA Barcoding of Thai Medicinal Plants, Department of Pharmacognosy and Pharmaceutical Botany, Faculty of Pharmaceutical Sciences, Chulalongkorn University, Bangkok 103300, Thailand; ⊥ National Electronics and Computer Technology Center (NECTEC), National Science and Technology Development Agency (NSTDA), Pathum Thani 12120, Thailand; # Synchrotron Light Research Institute, Nakhon Ratchasima 30000, Thailand

**Keywords:** SiO_2_-carbon core–shell, hollow carbon, thymol, DFT, antibacterial activity

## Abstract

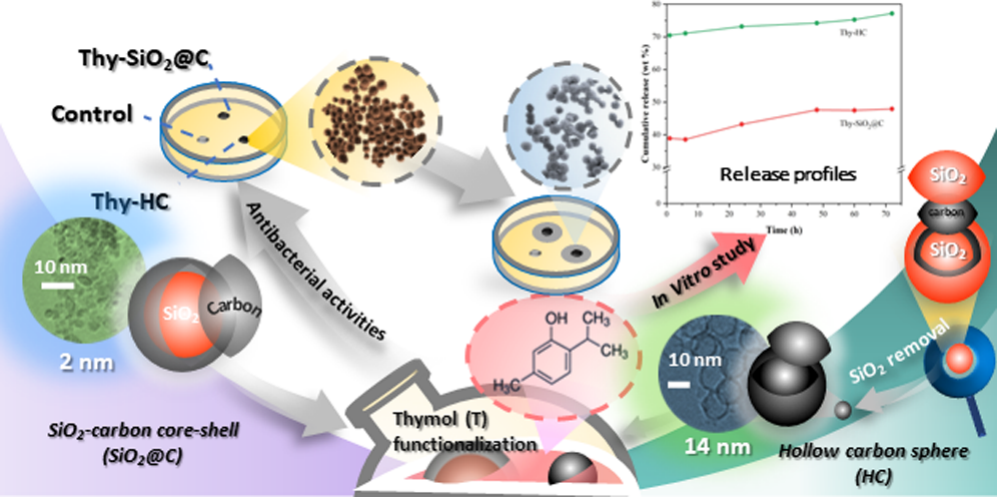

Functionalization of thymol (Thy) on nanocarriers is a key step in achieving prolonged antimicrobial activity. This requires nanomaterials with uniform particle diameters and suitable thymol sorption. Herein, hollow carbon (HC) and SiO_2_-carbon core–shell (SiO_2_@C) were investigated due to their diverse morphologies and ease of surface modification. HC (14 ± 1 nm size) and SiO_2_@C (10 ± 1.5 nm size) were synthesized by the Stöber method before thymol was loaded by incipient wetness impregnation. Nanoparticle physicochemical properties were characterized by advanced techniques, including X-ray photoelectron spectroscopy (XPS) and near-edge X-ray absorption fine structure (NEXAFS). Adsorption energies of thymol on the carbon and SiO_2_ surfaces were elucidated by density functional theory (DFT) simulations. Moreover, the *in vitro* thymol release profiles and antibacterial activity were evaluated. The experimental results indicated that the oxy-carbon surface species of HC led to longer thymol release profiles than the –OH group of SiO_2_@C. The DFT calculations revealed that the weaker physical interaction of thymol on HC was better for drug release than that on SiO_2_@C. Thus, a longer thymol release profile of HC with hollow structures showed better antibacterial performance against Gram-positive bacteria *Staphylococcus aureus* than that of SiO_2_@C with core–shell structures. This work confirms the important role of carbon morphology and specific functional groups in thymol release profiles for the further development of inhibition products.

## 1 Introduction

Silica (SiO_2_) nanoparticles have been widely used as drug-delivering materials. However, a desirable rate of drug release is difficultly controlled by using a single SiO_2_ support. Recently, carbon nanomaterials, including carbon nanotubes (CNTs),^1^ SiO_2_-carbon core–shell (SiO_2_@C),^2^ carbon dots (CDs),^3^ graphene oxide nanoblades,^4^ nanodarts,^5^ and hollow carbon (HC),^6^ have become preferable candidates for antibacterial applications because of their surface functional groups, intrinsic chemical inertness, and physical robustness.^7^ Among the different kinds of these nanosized carbons mentioned earlier, carbon-based nanospheres have drawn the most attention because their shapes and sizes can be tuned by adjusting synthesis conditions to achieve suitable drug carriers for medical and pharmaceutical applications.^8−10^

For instance, curcumin-derived CDs with quaternary ammonium species exhibited strong attachment to the cell membrane of the porcine epidemic diarrhea virus, resulting in a loss of integrity and death.^3^ Rose petal-derived CD nanocarriers with thymol reduced arthritic activity in rats.^11^ In addition, vancomycin-loaded HC provided better inhibition of *Escherichia coli* and *Staphylococcus epidermidis*, compared to both free vancomycin and its parent one.^6^ Although these carbon nanospheres show superior bacterial inhibition, their morphological effects, e.g., spheres of the same size with different structural properties and surface functionalities, have rarely been compared.

In the past, Beranová et al.^12^ clarified the morphological impact of diamond nanoparticles (DNPs), 5–50 nm in size, on a Gram-negative bacterium (*E. coli*) and a Gram-positive bacterium (*Bacillus subtilis*). The DNPs of 5 nm size were most effective against *E. coli*, while the DNPs of 18–50 nm sizes had higher antibacterial activity against *B. subtilis*. Norouzi et al.^13^ reported how size, surface chemistry, and the aggregation behavior of nanodiamonds of 18–125 nm sizes affected the inhibition of *E. coli*, *Staphylococcus aureus*, and *S. epidermidis*. The smallest nanodiamond at 500 μg/mL inhibited the growth of both *S. aureus* and *S. epidermidis* in a full bacterial medium. However, none of the nanodiamonds terminated the colony-forming ability of *E. coli* due to their low surface affinity for attachment on bacterial surfaces. Recently, Zhang et al.^7^ studied the effects of carbon-like diamonds (CLDs), including micro-, nano-, and ultrananocrystalline sizes, on their antibacterial activity against *E. coli* and *B. subtilis*. Among these materials, the CLD sample with microcrystalline-sized particles showed significant antibacterial activity against both *E. coli* and *B. subtilis*. Hence, this reveals that the carbon morphology influences the antibacterial properties.

Although previous research has shown that carbon nanospheres containing various antibacterial agents are active, their interfacial interactions have rarely been evaluated by a combination of experimental and theoretical methods. To the best of our knowledge, there has been no report comparing the morphological effect of carbon nanospheres with thymol on the inhibition of the same bacteria. Consequently, the aim of this work is to prepare carbon nanospheres with different morphologies, including hollow carbon and SiO_2_@C core–shell structures, and to modify their surfaces with thymol by incipient wetness impregnation. The adsorption of thymol on the nanosphere surfaces was confirmed with a combined experimental and density functional theory (DFT) study. In addition, both *in vitro* thymol release tests and antibacterial performance against *S. aureus* were also performed. This investigation not only demonstrates precise protocols for assessing materials with targeted adsorption properties but also provides new insights into the adsorption behavior of an alternative material for future nanocarrier applications.

## 2 Results and Discussion

### 2.1 Characterization of Carbon Nanospheres

**Figure 1:**
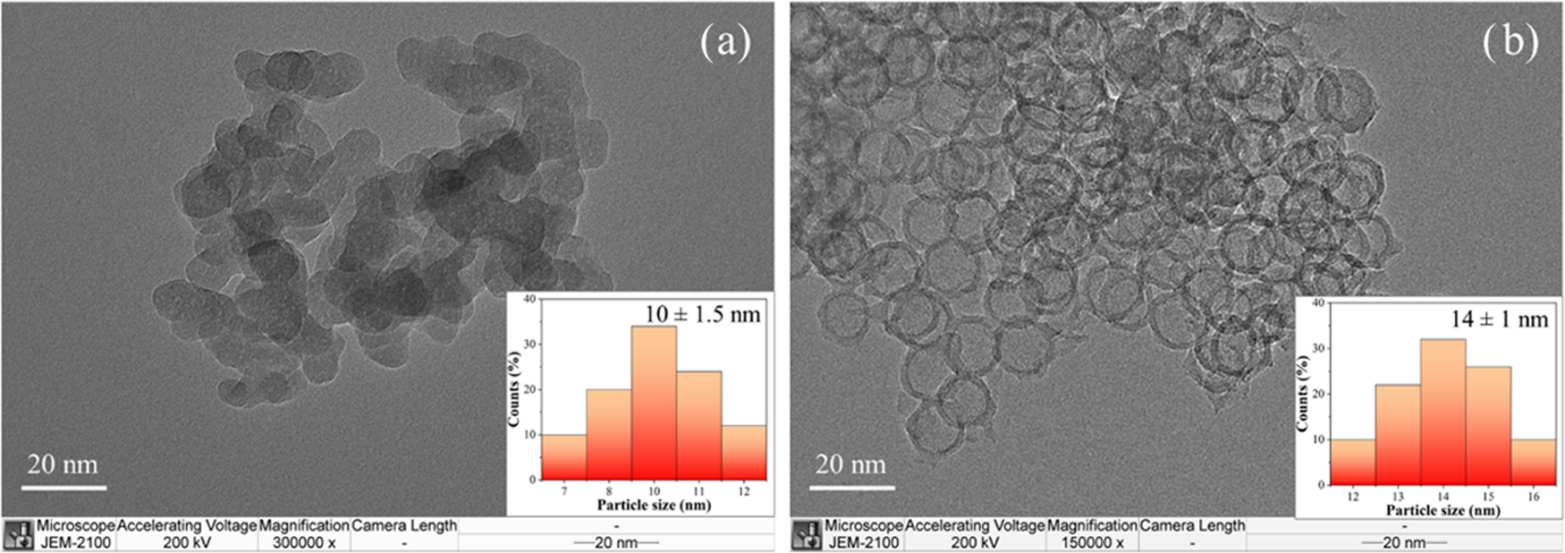
TEM image with the particle size distribution (inset) of (a) SiO_2_@C nanospheres and (b) hollow carbon.

Transmission electron microscopy (TEM) images and the particle size distributions of SiO_2_@C and HC are shown in Figure 1a,b, respectively. The SiO_2_@C core–shell is irregularly spherical, with an average particle size of 10 ± 1.5 nm (see the inset) and some agglomeration. The carbon shell of the core–shell nanosphere could be rarely observed by TEM, possibly due to its ultrathin carbon-shell thickness according to the previous observation by Niu et al.^14^ A hollow structure is observed for the HC nanosphere, with an average particle size of 14 ± 1 nm, corresponding to a previous report by Zhao et al.^8^ Comparing the carbon wall thickness, the micrographs suggested that the wall structure of HC was significantly thicker than that of the core–shell. These results confirmed that carbon nanospheres with different morphologies, including core–shell and hollow structures, were successfully prepared and ready to be used as nanocarriers by the incipient wetness impregnation of thymol.

**Figure 2:**
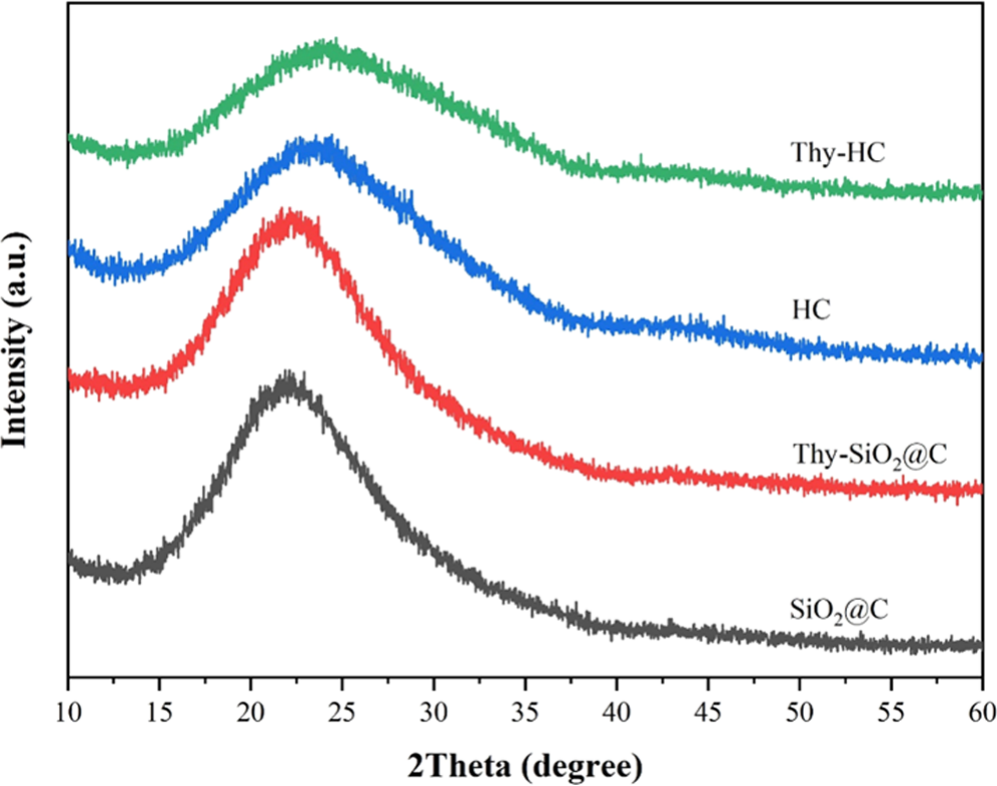
XRD patterns of carbon nanospheres with thymol compared to those of the parents.

X-ray diffraction (XRD) patterns of the obtained nanospheres with and without thymol are shown in Figure 2. The core–shell shows a broad peak at approximately 22°, related to a characteristic of amorphous carbon and silica.^15,16^ However, the XRD pattern of the HC sample shows a peak shift to ∼24°, corresponding to graphitic carbon (graphite).^8^ The result indicates that the HC sample achieved some carbon graphitization, while the core–shell did not, in agreement with the wall thickness in the TEM results. Additionally, the XRD patterns from both samples with thymol were similar to those of their parents, indicating the high stability of the nanosphere structures.

**Figure 3:**
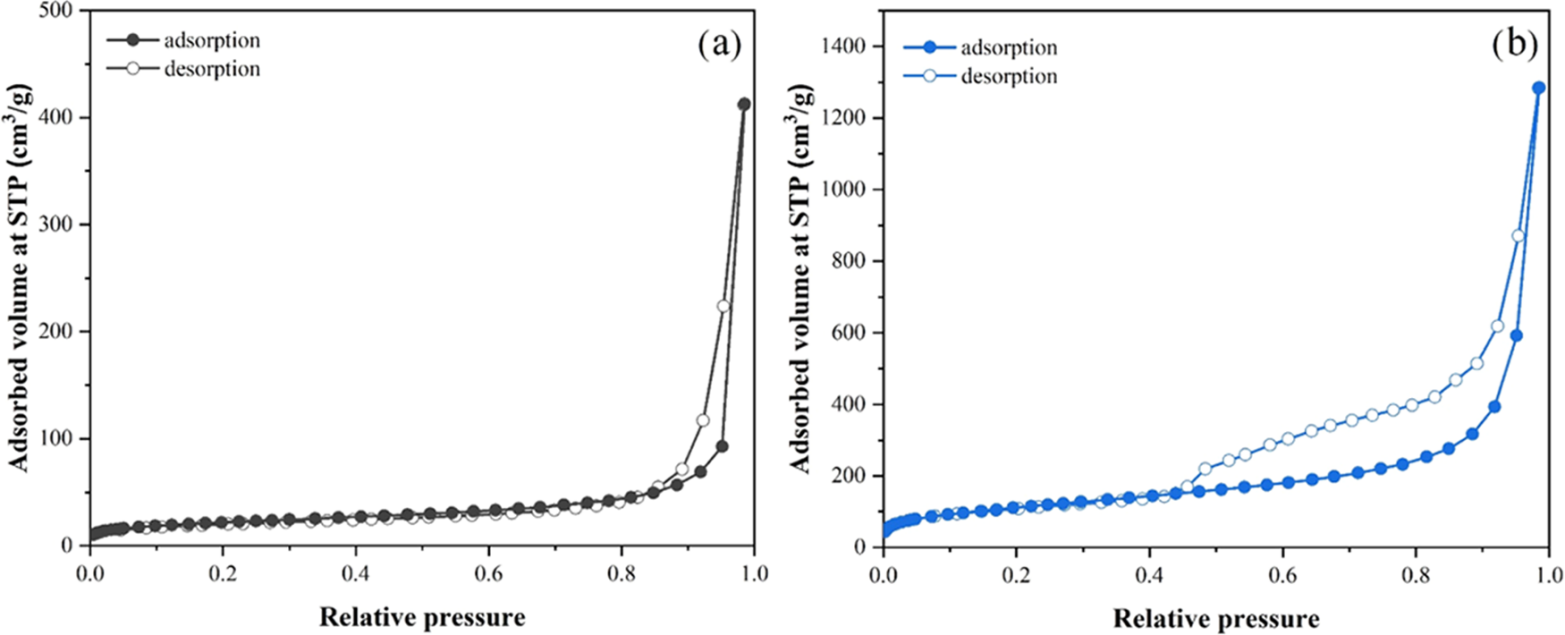
N_2_ adsorption–desorption isotherms of (a) SiO_2_@C nanospheres and (b) hollow carbon.

The N_2_ adsorption–desorption isotherms of all samples are shown in Figure 3. Both parent nanospheres exhibit a combined type I(b) and IV(a) isotherm according to the IUPAC classification, implying the appearance of micropores and mesopores with small external surfaces.^17,18^ The isotherms also show a type H4 hysteresis loop caused by micropore filling with N_2_ at a low relative pressure, suggesting agglomerated nanospheres of micromesoporous carbons,^17^ consistent with the TEM results. Remarkably, the size of the hysteresis loop from HC was larger than that from SiO_2_@C, implying a larger pore volume. In addition, the specific surface areas, nonlinear density functional theory (NLDFT) pore volumes, and diameters of carbon nanospheres are summarized in Table 1. The HC nanospheres had larger surface areas and pore volumes than the core–shell. However, HC had a larger pore diameter than the other samples, possibly due to the etching effect of base species during silica core removal. This result could suggest that the carbon nanospheres had distinctive surfaces and porosities and had a different mode of antibacterial activity after thymol impregnation.

**Table 1:** Specific Surface Areas, NLDFT Pore Volumes, and Diameters of the Carbon Nanospheres

sample	specific surface area^a^ (m^2^/g)	pore volume^b^ (cm^3^/g)	pore diameter^b^ (nm)
SiO_2_@C	76	0.11	2.58
HC	405	1.48	3.79

a Specific surface area was calculated with the Brunauer–Emmett–Teller (BET) method.

b Both pore volume and diameter were evaluated with the NLDFT method.

**Figure 4:**
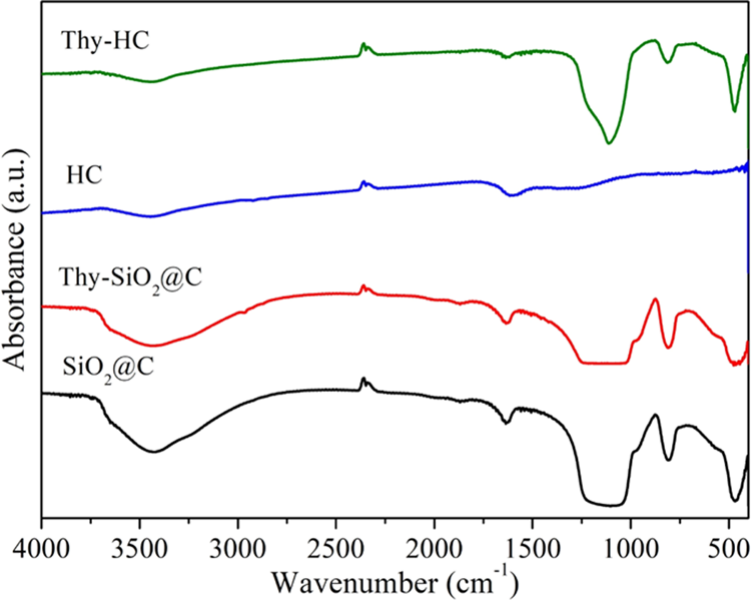
FTIR spectra of SiO_2_@C and HC with thymol compared to those of the parents.

The Fourier transform infrared (FTIR) spectra of SiO_2_@C and HC against their parent samples are depicted in Figure 4. SiO_2_@C showed vibration peaks at 1100–1080, 760, and 450 cm^–1^ assigned to the asymmetric and symmetric stretching and bending modes of Si–O–Si, respectively, as the characteristics of the silica core.^19^ After removing the silica core of HC by NaOH etching, these silica characteristics were completely invisible by FTIR, indicating the successful removal of the silica core.^20,21^ After the impregnation of thymol on the nanosphere surfaces, vibrational bands were observed at 1417 and 804 cm^–1^, related to the phenyl ring.^22^ This FTIR result indicated that thymol was successfully introduced onto the surfaces of the carbon-based nanospheres.

**Figure 5:**
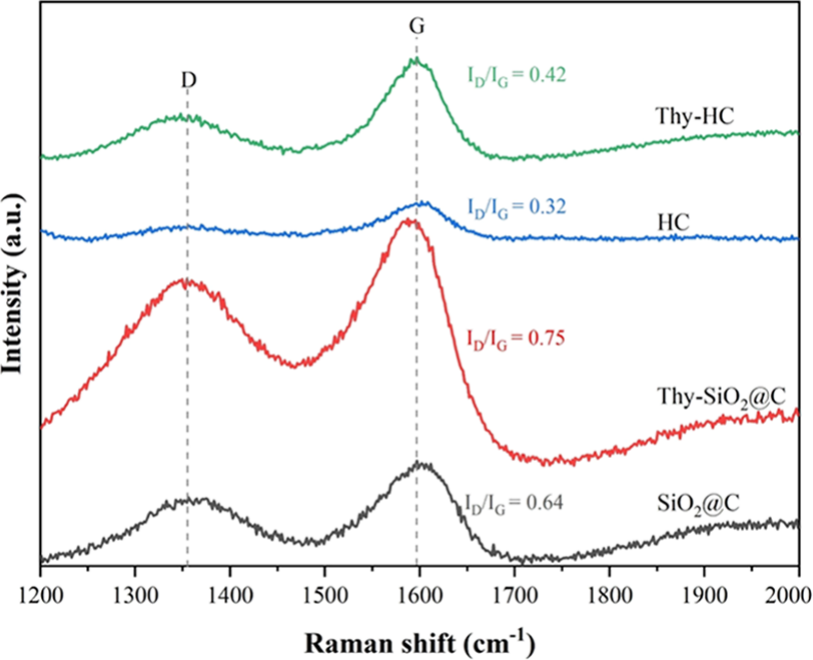
Raman spectra of carbon nanospheres with thymol compared to their parents.

To further ensure the appearance of the carbon structure from both core–shell and hollow structures, the Raman spectra of the nanospheres with and without thymol loading are collected and shown in Figure 5. All samples show two intense peaks at approximately 1355 and 1600 cm^–1^, corresponding to the D band from structural disorder and the G band from graphitic carbon with sp^3^ and sp^2^ hybridization.^23^ Moreover, the intensity ratio between the D and G bands (*I*_D_/*I*_G_) can define the degree of graphitization for these carbon nanospheres.^24^ A comparison of both parents shows that HC has a ratio half that of SiO_2_@C, indicating a higher degree of graphitization.^25^ Interestingly, the G band from both samples with thymol shows a red shift, namely, from 1602 to 1590 cm^–1^ for Thy-SiO_2_@C and from 1605 to 1597 cm^–1^ for Thy-HC, possibly due to their surface interactions with thymol molecules. In addition, they had a higher *I*_D_/*I*_G_ value than their parents, suggesting an increase in defect states in the sp^2^ graphene planes.^25^ Thus, the presence of thymol moieties could alter the degree of graphitization of these carbon nanospheres.

**Figure 6:**
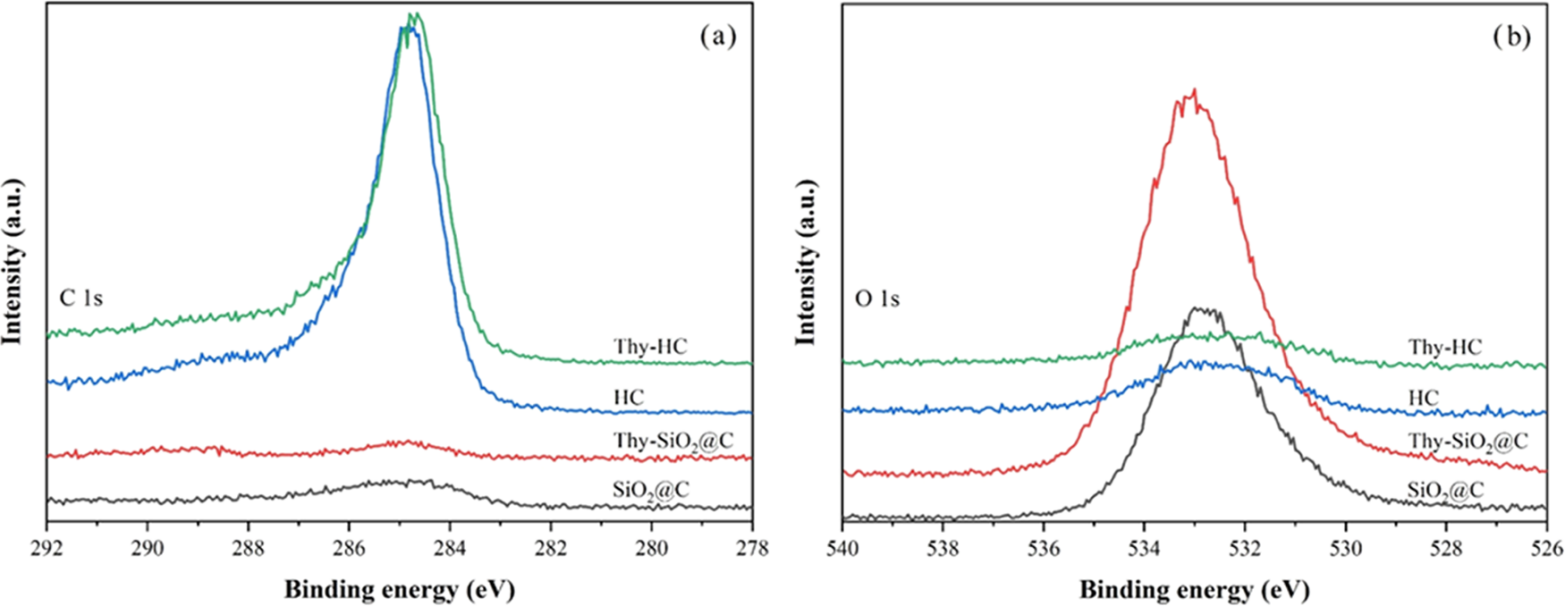
XPS spectra of carbon nanospheres impregnated with thymol compared to the parent core–shells: (a) C 1s and (b) O 1s spectra.

To further elucidate the chemical species on the sample surfaces, XPS analysis was employed. The C 1s and O 1s XPS spectra from all samples are depicted in Figure 6a,b, respectively. The HC samples display an intense C 1s peak rather than another peak due to a thicker carbon wall and higher graphitic carbon based on the TEM and Raman results, respectively. In contrast, the O 1s peak intensity from SiO_2_@C is higher than that from HC, which was possibly the result of either the SiO_2_ core or surface oxygen species. Based on the results of deconvoluted C 1s and O 1s XPS peaks, the binding energies (BE) and peak area percentages of all surface species are summarized in Table 2. These suggested surface species on the carbon nanospheres containing C–C/C–H, C–O, C=O, and C–O–Si moieties (only belonging to SiO_2_@C).^10,18,26,27^ Satellite peaks could be found in all carbon samples. The C–O–Si and Si–O–H species from the core–shell are observed at approximately 291 and 535 eV, respectively, indicating interfacial bonding between the carbon shells and the SiO_2_ core.^27^ However, the C 1s peak area of the C–C/C–H species from HC is higher than that from the core–shell because of a higher graphitic carbon content. After thymol loading, both the C 1s and the O 1s peaks shifted slightly to higher binding energies. This could be from the interfacial interaction of thymol molecules with the sp^2^-hybridized carbon and available oxygen functional groups, in agreement with the Raman results. Therefore, the finding confirmed the presence of thymol molecules on the sample surfaces.

**Table 2:** Comparisons of Carbon Nanospheres with and without Thymol Were Based on Binding Energies and Peak Areas from Deconvolution of the XPS Spectra

	SiO_2_@C		Thy-SiO_2_@C		HC		Thy-HC		ref
surface species	BE (eV)	area (%)	BE (eV)	area (%)		BE (eV)	area (%)	BE (eV)	area (%)
C 1s									
C–C/C–H	284.59	47.92	284.89	50.49	284.81	72.24	284.86	78.88	(10,18,26)
C–O	286.18	23.66	286.79	7.79	286.32	16.57	286.47	14.66	(10,18,26)
C=O	288.37	15.17	289.12	29.72	288.04	6.63	288.08	5.99	(10,18,26)
C–O–Si/satellite	291.31	13.25	291.10	12.00	289.56	4.55	289.67	4.46	(27)
O 1s									
C=O	530.93	12.74	530.95	10.12	531.80	47.40	531.82	49.97	(10,26)
C–O–C/C–O–H/Si–O–Si	532.85	85.99	533.08	88.87	533.44	52.60	533.61	50.03	(10,18,26)
Si–O–H/adsorbed H_2_O	535.38	1.27	536.32	1.01	nd^a^	nd	nd	nd	(18,26)

a nd = Not detectable.

**Figure 7:**
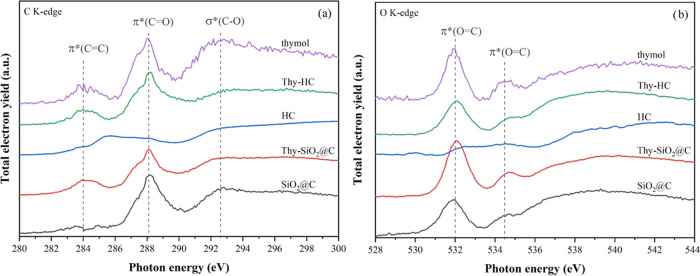
NEXAFS of (a) C K-edge and (b) O K-edge spectra for SiO_2_@C and HC with and without thymol.

In addition, the chemical states associated with local electronic structures were determined with NEXAFS. The C and O K-edge spectra and peak assignments for the nanospheres with and without thymol are shown in Figure 7a,b, respectively. Both core–shells show the C K-edge peaks at approximately 284 and 292 eV, corresponding to the transition states of 1s → π* (C=C)^28^ and 1s → σ* (C–O/C–C),^29^ respectively. Moreover, the peak at ∼288 eV from the 1s → π* (C=O) transition^28^ is from amorphous carbon.^30^ In comparison, the parent HC shows an intense peak at approximately 285.4 eV, assigned to the 1s → π* (C=C) transition, which originated from graphitic carbon,^30^ while the peak for amorphous carbon at 288 eV is rarely observed. This verified that the HC sample had graphitic carbon rather than SiO_2_@C. However, the amorphous carbon peak from Thy-HC increased with thymol loading. In addition, the O 1s → π* (O=C) transition of both the O K-edge peaks^31^ from Thy-SiO_2_@C and Thy-HC (see Figure 6b) increased with thymol loading. These results confirmed that thymol was located on the sample surfaces, consistent with the Raman and XPS results.

### 2.2 *In Vitro* Thymol Release Profile

**Figure 8:**
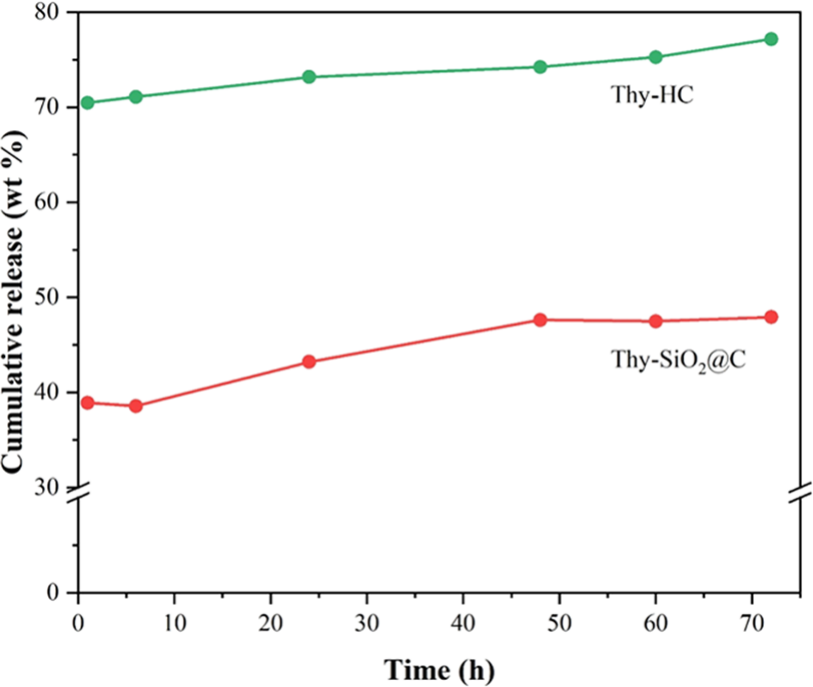
Thymol release profiles for Thy-SiO_2_@C and Thy-HC, determined in phosphate buffer (pH 7.4) at 25 °C.

The thymol release profile from both Thy-SiO_2_@C and Thy-HC via the *in vitro* method in phosphate buffer (pH 7.4) at 25 °C with different time intervals is shown in Figure 8. More interestingly, the Thy-HC sample quickly reached a cumulative thymol release of ∼70 wt % within an hour, while another sample showed approximately 40 wt %. When the release time was extended to 72 h, the obtained release from Thy-SiO_2_@C approached 50 wt %. The observation suggested that the hollow structure consisting of only carbon functional groups (with a higher surface area) could provide faster thymol desorption than the core–shell structure with a combination of carbon and silica species. The surface interaction and adsorption energy between the carbon nanospheres and thymol moieties were further investigated with the DFT method, and their inhibitory activity was also evaluated.

### 2.3 Adsorptive Interactions of Thymol on Silica and Carbon Surfaces

**Figure 9:**
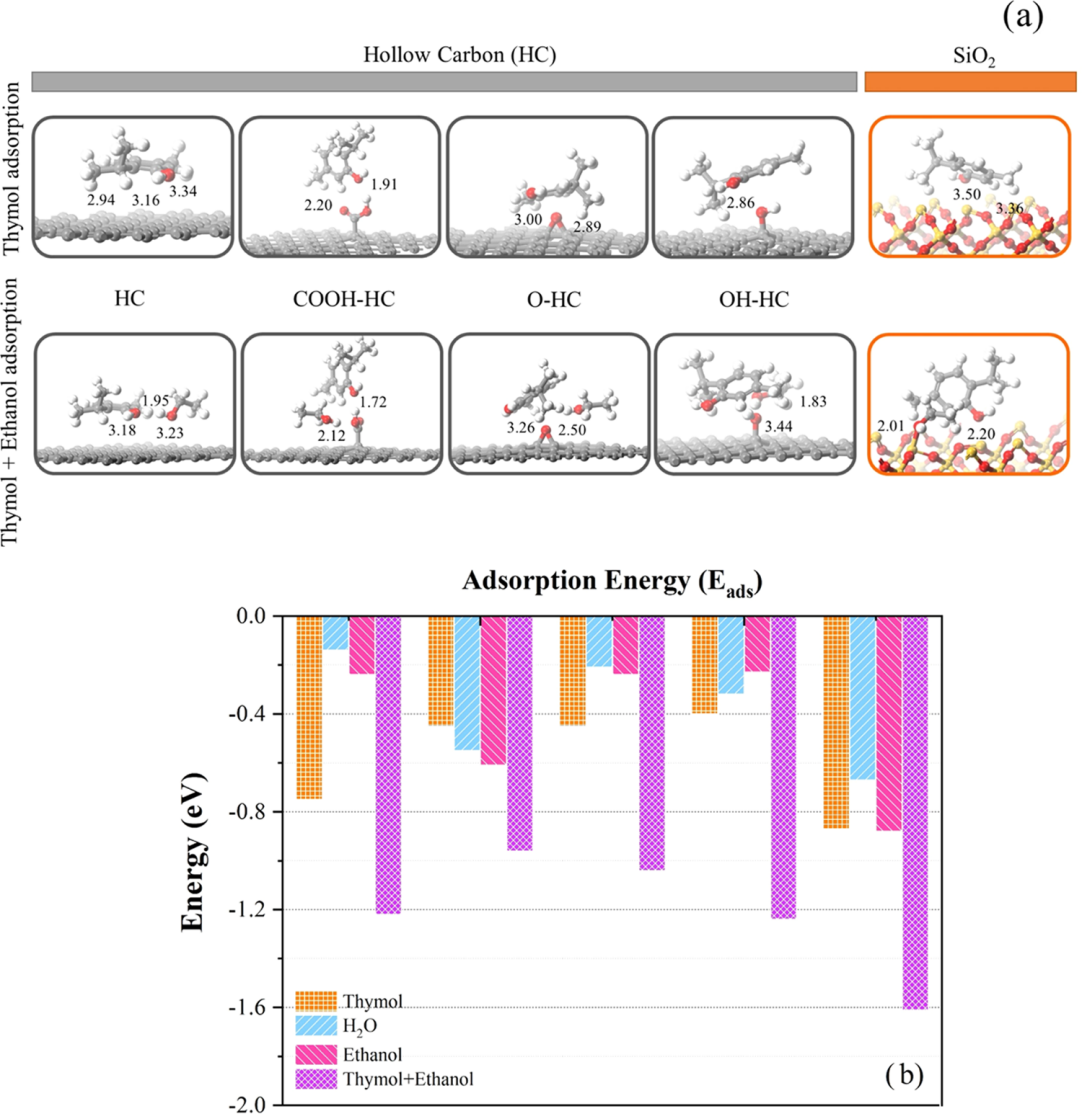
(a) Geometrical optimizations of thymol adsorption on the HC and SiO_2_ surfaces. (b) Adsorption energy diagram of thymol, H_2_O, ethanol, and coadsorbed thymol and ethanol on the HC and SiO_2_ surfaces.

To confirm the behavior and/or mechanism of thymol adsorption on the HC surface, we modeled the surface of HC by the decoration of hydroxy (–OH), carbonyl (–COOH), and epoxy (-O–) groups on a graphene-like sheet in DFT calculations. The adsorption energies for thymol adsorption on each model are illustrated in Table 3. Our calculations revealed that the interactions between thymol and the carbon surfaces without functional groups involved physisorption with *E*_ads_ values of −0.75 eV for the π–π interactions of the aromatic carbon surface and the six-membered ring of thymol. The distances between the thymol arene rings and the HC surfaces are approximately 2.94 and 3.34 Å, as demonstrated in Figure 9. In addition, the adsorption mechanism involving π–π interactions between the six-membered ring of thymol and the functionalized hydroxy (–OH) and epoxy (-O–) groups on the HC surfaces was found to have *E*_ads_ values of −0.45 and −0.40 eV, respectively. In contrast, thymol adsorption via hydrogen bonding between the OH group of thymol and the –COOH group of HC was observed with distances of 1.91 and 2.20 Å, respectively. Moreover, we compared thymol adsorption on silica surfaces. Remarkably, SiO_2_ has a greater thymol adsorption strength than HC surfaces, with an adsorption energy of −0.87 eV, which is in good agreement with the thymol release profiles.

**Table 3:** Adsorption Energy (*E*_ads_) of Thymol, H_2_O, Ethanol, and Coadsorbed Thymol and Ethanol on the HC and SiO_2_ Surfaces

	adsorption energy (eV)				
models	HC	COOH-HC	O-HC	OH-HC	SiO_2_
thymol adsorption	–0.75	–0.49	–0.45	–0.40	–0.87
H_2_O adsorption	–0.14	–0.55	–0.21	–0.32	–0.67
ethanol adsorption	–0.24	–0.61	–0.24	–0.23	–0.88
thymol and ethanol coadsorption	–1.22	–1.00	–1.04	–1.24	–1.61

Moreover, the coadsorption of thymol and ethanol was investigated to determine the effect of thymol adsorption in the presence of ethanol. The *E*_ads_ values on HC are in the range of −1.00 to −1.24 eV, while the adsorption on SiO_2_ is found to yield −1.61 eV. The results showed that the coadsorption energy on SiO_2_ is higher than that on the HC surfaces, corresponding to the results of thymol adsorption. Interestingly, the coadsorption energy on either HC or SiO_2_ is higher than that of single-molecule adsorption due to the H-bonding interactions between thymol and ethanol.

### 2.4 Antibacterial Activity of Thymol-Impregnated Carbon Nanospheres

**Figure 10:**
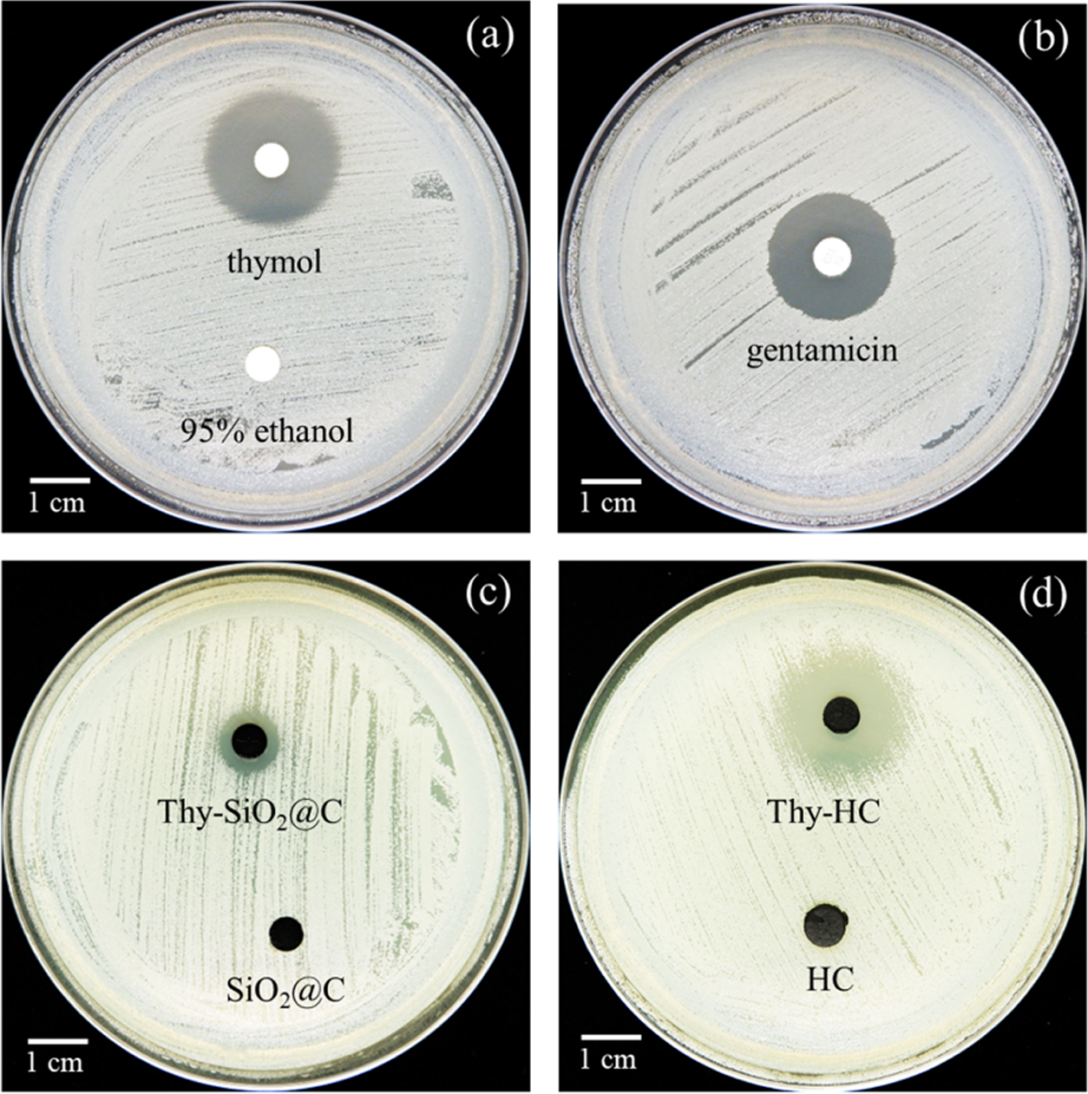
Antibacterial performance of carbon nanospheres with and without thymol against *S. aureus* compared to the control. Inhibition zones with (a) thymol and 95% ethanol, (b) gentamicin, (c) SiO_2_@C and Thy-SiO_2_@C, and (d) HC and Thy-HC after 20 h of incubation.

A qualitative assessment of *in vitro* antibacterial activity against *S. aureus* ATCC 25923, a Gram-positive bacterium, was performed based on measurements of the inhibition zones of the carbon nanospheres with and without thymol. The results are depicted in Figure 10 and summarized in Table 4. Both free thymol and gentamicin had wider inhibition zones against *S. aureus* than 95% ethanol, and the parents, SiO_2_@C and HC (see Figure 10c,b), were inactive. Although both the carbon nanospheres with thymol were active, the inhibition zone of Thy-HC was wider than that of Thy-SiO_2_@C (see Table 4). The result indicated that the carbon with a hollow structure type was a better carrier of thymol than that with a core–shell structure due to a larger surface area and suitable physisorption between thymol and the surface, based on the *in vitro* release profiles and the DFT study. Thymol and released thymol showed clear inhibition zones against *S. aureus*. This phenomenon might be due to the antibacterial mechanism of thymol that directly disrupted the outer and inner membranes as well as destabilized the DNA, leading to a depletion of protein expression and an increase in bacterial cell death.^32−36^ This finding confirmed that the carbon nanospheres with thymol from this work were sufficiently active against *S. aureus* and were comparable to carbon-based materials reported elsewhere.^22,37,38^ In addition, all of the results showed promising beneficial materials for wound dressing applications. However, fabrication processes as well as *in vivo* assessments of biocompatibility are still needed.^39−42^ Therefore, carbon nanospheres should be further developed and utilized in preventive healthcare and medical devices, e.g., as drug nanocarriers and inhibition products.

**Table 4:** Diameter of Inhibition Zones Against *S. aureus* of Thymol, SiO_2_@C, and HC with and without Thymol

samples	diameter of inhibition zones (mm ± SD)
SiO_2_@C	nd^a^
Thy-SiO_2_@C	10.12 ± 1.06
HC	nd^a^
Thy-HC	18.93 ± 0.03
thymol	23.73 ± 0.24
gentamicin	22.81 ± 0.15
95% ethanol	nd^a^

a nd = An inhibition zone was not detectable.

## 3 Materials and Methods

### 3.1 Chemicals

All chemicals were commercially available and used as received without any purification, including 3-(aminopropyl)triethoxysilane (APTES, NH_2_C_3_H_6_Si(C_2_H_5_O)_3_, 98.0%, Sigma-Aldrich), ammonia solution (NH_3_.H_2_O, 28–30%, RANKEM), anhydrous ethanol (C_2_H_5_OH, 99.9%, RCI Labscan), anhydrous disodium hydrogen phosphate (Na_2_HPO_4_, 99.5%, EMSURE, Merck), Brij56 ((C_2_H_4_O)_n_.C_16_H_34_O, molecular weight 623, Spectrum Chemical MFG Corp), cyclohexane (C_6_H_12_, 99%, Sigma-Aldrich), ethylene carbonate ((CH_2_O)_2_CO, 99.0%, TCI), isopropanol (C_3_H_7_OH, 99.8%, Fisher Chemical), orthophosphoric acid (H_3_PO_4_, 85%, LobaChemie Pvt. Ltd.), potassium chloride (KCl, 99.5%, EMSURE, Merck), potassium dihydrogen phosphate (KH_2_PO_4_, 99.5%, EMSURE ISO, Merck), sodium chloride (NaCl, 99.5%, EMSURE ACS, ISO, Merck), sodium hydroxide pellets (NaOH, 99.0%, EMSURE, Merck), sulfuric acid (H_2_SO_4_, 98%, RCI Labscan), tetraethyl orthosilicate (TEOS, Si(OC_2_H_5_)_4_, 98%, Thermo Scientific), and thymol (C_10_H_14_O, 98.0%, TCI).

### 3.2 Nanosphere Synthesis and Incipient Wetness Impregnation with Thymol

A nanosized SiO_2_@C core–shell was synthesized using the 20 v/v % ethanol solution, as described in detail from our previous report.^2^ The as-prepared HC sample was synthesized according to a previous report.^8^ Brij56 (2.12 g) and cyclohexane (7.5 mL) were stirred at a rate of 400 rpm at 50 °C in a two-neck round-bottom flask equipped with a condenser. After a clear solution was obtained, deionized water (0.2 mL) was injected under stirring. Ammonia solution (0.57 mL) and TEOS (0.2 mL) were added in sequence, and the mixture was stirred for another 2 h. APTES (50 μL) was then injected into the clear solution, and the mixture was stirred overnight. To prepare a resin precursor, resorcinol (0.25 g), formaldehyde (0.35 mL), and ethanol (3 mL) were mixed and stirred for 10 min at room temperature. The resin precursor (1 mL) and ammonia solution (0.8 mL) were then added dropwise, and the mixture was further stirred for 24 h at 50 °C. Subsequently, TEOS (2 mL) was slowly dropped to the suspension, and the mixture was stirred for another 12 h. The obtained dark brown resultant was centrifuged, washed with 2-propanol and water, and freeze-dried in a CHRIST laboratory freeze-dryer at −60 °C for 24 h. The obtained powder was carbonized with a Nabertherm high-temperature tube furnace at 600 °C for 3 h with a N_2_ flow rate of 10 mL/min. Finally, the resulting black powder was etched with 2 M NaOH (50 mL) at 50 °C for 24 h to remove silica. The black resultant mixture was washed with water and freeze-dried at −60 °C for 24 h.

Incipient wetness impregnation of HC and SiO_2_@C core–shell with thymol (Thy) solution (10 g/L) was carried out according to our previous literature.^2^ The HC and SiO_2_@C samples impregnated with thymol were called Thy-HC and Thy-SiO_2_@C, respectively.

### 3.3 Material Characterization

The shape and particle size were measured by high-resolution transmission electron microscopy (JEM 2100, JEOL) at a voltage of 200 kV. Structural characteristics based on X-ray diffraction (XRD) patterns were obtained with a Bruker D8 advanced X-ray diffractometer using Cu K_α_ radiation operating at a voltage of 40 kV and a current of 40 mA. The specific surface area, pore volume, and diameter were measured by nitrogen sorption with a NOVA e-Series instrument operating at −196 °C. The predried sample powder was degassed at 250 °C for 3 h. The specific surface area and pore size were calculated with the Brunauer–Emmett–Teller (BET) method, and the volume was calculated with nonlinear density functional theory (NLDFT) methods.

The functional groups of all of the samples were analyzed by an NT-MDT confocal Raman spectrometer using an excitation wavelength of 633 nm, ranging from 50 to 2000 cm^–1^ with a scan number of 12 and a scan rate of 10 s/scan. At beamline BL5.3 (SLRI-NANOTEC-SUT), X-ray photoelectron spectroscopy (XPS) was used to identify surface species using a ULVAC-PHI PHI5000 VersaProbe II with Al K_α_ radiation. The XPS spectra were performed with CasaXPS software and calibrated with the C 1s peak at 284.8 eV. The XPS peak deconvolution was obtained using Shirley background subtraction and Gaussian–Lorentzian line shape combinations. At beamline BL3.2 in the partial electron yield mode, chemical states were studied by near-edge X-ray absorption fine structure (NEXAFS). Highly oriented pyrolytic graphite was used to calibrate the K-edge spectra of both C and O atoms. Both beamlines were at the Synchrotron Light Research Institute, Nakhon Ratchasima, Thailand.

### 3.4 *In Vitro* Thymol Release Study

An *in vitro* thymol release study of the prepared nanospheres was performed with the method from our recent work.^2^ In brief, 66.7 w/v % of the sample in the mixed solution of phosphate-buffered saline (PBS, pH ∼ 7.4) and 40 v/v % ethanol was shaken at 25 °C with various operation times. Finally, the releasing thymol in the filtered solution was analyzed by using a PerkinElmer Lambda 650 UV–vis spectrophotometer at a wavelength of 275 nm. The cumulative thymol release (wt %) was obtained from eq 1.
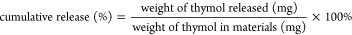
1

### 3.5 DFT Method and Models

DFT calculations were performed with the Vienna ab initio simulation package (VASP). The electron exchange–correlation was represented by the Perdew–Burke–Ernzerhof (PBE) functional with generalized gradient approximation (GGA).^44^ The interaction between ion cores and valence electrons was assigned by the projector augmented wave (PAW) method,^45^ and we used Grimme’s dispersion correction (DFT-D3)^46^ to account for the van der Waals force. The kinetic energy cutoff for plane wave expansions was set to 450 eV, and the reciprocal space was sampled by using 3 × 3 × 1 *k*-point grids in Monkhorst–Pack meshes for structural optimization. The convergence criteria were 1 × 10^–5^ eV energy differences for the electronic wave function for structure optimization calculations. All atomic coordinates were converged to within 0.02 eV/Å for the maximal components of forces.

We modeled a 7 × 7 supercell lattice of a hollow carbon structure containing 98 carbon atoms called hollow carbon (graphene-like sheet). As seen in Figure 9, the optimized lattice parameters were *a* = *b* = 2.46 Å, corresponding to the experimental values.^47^ To simulate the surface of the HC model, functional groups such as hydroxy (–OH), carbonyl (–COOH), and epoxy (–O–) were applied to the graphene-like sheet. The structure of the bulk SiO_2_ was also simulated. Then, the SiO_2_ (001) plane was sequentially modeled by a 2 × 2 periodic supercell (80 atoms) composed of a two-layer slab to represent a silica surface. The vacuum space was set to 15 Å to separate the layers and prevent interaction between the layers. The adsorption energy (*E*_ads_) of thymol was computed from eq 2.
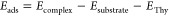
2where *E*_complex_ is the energy of the adsorption complex. *E*_substrate_ and *E*_Thy_ are the energies of the substrate and the individually adsorbed thymol, respectively. A higher negative value of *E*_ads_ indicates stronger thymol adsorption.

### 3.6 *In Vitro* Antibacterial Activity Assays

*In vitro* antibacterial activity assays by the Kirby–Bauer disk diffusion susceptibility test method^49^ against *S. aureus* ATCC 25923 as a Gram-positive bacterium was studied using a procedure from our recent report.^2^ The *S. aureus* suspension was spread onto Mueller–Hinton agar (Difco). The solution of thymol in ethanol (6 w/v %) was dropped into a sterile paper disk (Sigma-Aldrich) with a 6 mm diameter. Ethanol was dropped onto a paper disk as the control. A standard gentamicin disk (BD BBL) was used as a positive control. These prepared disks were placed on an inoculated agar plate and kept at 37 °C for 20 h. After the course of testing, the obtained inhibition zones were determined with a Mitutoyo Vernier caliper.

## 4 Conclusions

Carbon nanospheres with different morphologies, including hollow and core–shell structures, were successfully prepared and modified with thymol by incipient wetness impregnation. Hollow carbon (HC, 14 ± 1 nm) had a larger surface area and a higher degree of graphitization than core–shell carbon (SiO_2_@C, 10 ± 1.5 nm). In the *in vitro* thymol release test, HC showed 70 wt % release within an hour, while the rest approached 50 wt % at 72 h. The DFT studies revealed that the weak physisorption of the thymol moiety on the HC surface resulted in faster and easier desorption. Remarkably, thymol-containing HC exhibited a better inhibitory behavior against *S. aureus* than the other thymol additives. Therefore, carbon nanospheres are promising candidates for inhibition products for medical and pharmaceutical applications.
